# Breast Cancer Diagnosis and Management in an Area of Protracted Conflict: A Case Series from Northwest Syria

**DOI:** 10.1055/s-0045-1802585

**Published:** 2025-05-19

**Authors:** Mohamed Hamze, Jude Alawa, Fares Alahdab, Alaa Al-Shemali, Ahmed Najeb Alhussein, Nour Muhammed Ali Arab, Bayan Galal, Jamil Debel, Ayham Jemo, Molham khalil, Anees Chagpar, Bassel Atassi, Kaveh Khoshnood, Aula Abbara

**Affiliations:** 1 Department of Research, Syrian American Medical Society, Washington DC, United States; 2 School of Medicine, Stanford University, Stanford, California, United States; 3 Department of Biomedical Informatics, Biostatistics, Epidemiology, University of Missouri-Columbia, Columbia, Missouri, United States; 4 Department of Medicine, Division of Cardiovascular Medicine, University of Texas Health Sciences Center at Houston, Houston, Texas, United States; 5 Faculty of Medicine, Free Aleppo University, Aleppo, Syria; 6 Perelman School of Medicine, University of Pennsylvania, Philadelphia, Pennsylvania, United States; 7 Department of Oncology - Idlib National Hospital , Syrian American Medical Society, Idlib, Syria; 8 Department of Surgery, Yale University, New Haven, United States; 9 Chicago Medical School - OSF Little Company of Mary Medical Center, Rosalind Franklin University of Medicine and Science, North Chicago, Illinois, United States; 10 Oncology Committee, Syrian American Medical Society, Washington DC, United States; 11 Department of Epidemiology of Microbial Diseases, Yale School of Public Health, New Haven, Connecticut, United States; 12 Department of Infectious Diseases, Imperial College, London; 13 Research Committee, Syrian American Medical Society, Washington DC, United States

**Keywords:** armed conflict, breast cancer, clinical presentation, northwest Syria, oncology service

## Abstract

**Background** Breast cancer remains a significant public health challenge in conflict-affected regions. This study aims to investigate the impact of armed conflict on the burden of breast cancer in female patients in northwest Syria, focusing on clinical presentations, management, diagnosis, access to care, and treatment outcomes.

**Methods** We conducted a retrospective analysis of breast cancer patients diagnosed at the Idlib Oncology Center between March 2017 and January 2022. Data were extracted from clinical files and analyzed in R. The study was conducted at the Idlib Oncology Center, the main referral center for cancer care in northwest Syria, serving a population of 4.6 million.

**Results** A total of 192 patients were included, with a median age of 45.5 years (interquartile range [IQR]: 40–56). Of 108 patients, 56.5% were internally displaced. Most patients were diagnosed with invasive ductal carcinoma (81.3%), and the majority presented at stages II and III (34.8 and 59%, respectively). Among 192 patients, 95.8% underwent surgery, with 96.6% having a mastectomy and 90.7% receiving chemotherapy. The median interval from symptom onset to diagnosis was 100.5 days, that from diagnosis to surgery was 14.5 days, and that from surgery to radiotherapy was 229 days. No significant effect was observed for chemical weapon exposure or family loss on survival. Displacement was associated with significantly lower predicted survival ( *p* = 0.0038; 95% confidence interval [CI]: 0.05064–0.2570).

**Conclusions** This study highlights a high prevalence of late-stage breast cancer, a high rate of mastectomies, delayed access to radiotherapy, and long delays between diagnosis and treatment in northwest Syria. Displacement negatively affects survival rates. Additionally, the substantial lack of radiotherapy in patients needing it and prolonged intervals between treatments contribute to poorer outcomes. Establishing localized oncology services and increasing funding for cancer medications and radiotherapy would improve access to necessary oncology care in this region.

## Introduction

Cancer is a growing burden in low- and middle-income countries (LMICs), with breast cancer being a particular concern as it is currently the most commonly diagnosed cancer worldwide, with an estimated 2.3 million new cases annually. 1 When breast cancer is diagnosed and treated early, the survival rate after 5 years exceeds 90%. 2 Women in LMICs often present with more advanced diseases and have worse outcomes compared to those in high-income countries. 3 This is further exacerbated in regions experiencing conflicts, where the impact of factors such as changes in risk exposure, behavioral changes, and limited access to timely health care is exacerbated. 4 Breast cancer outcomes are particularly affected, as women in these settings may face delays in, or complete lack of, screening, diagnosis, and treatment, leading to worse survival rates. Additionally, conflicts can increase exposure to toxic and chemical substances, potentially influencing the development and progression of breast cancer. 5

In Syria, the protracted armed conflict has led to the emergence of multiple adaptive health systems, which have evolved under different jurisdictions. As such, published data generally do not comprehensively capture information on breast cancer from all conflict-affected areas. Reported data are mostly from hospital-based registries in areas under government control 6 or are extrapolated from information from neighboring countries. According to the WHO Global Cancer Observatory, Syria has an age-standardized incidence rate of 138.6 cases and an age-standardized mortality rate of 91.0 deaths per 100,000 people for all cancers. Breast cancer is the most commonly diagnosed cancer in Syria, with 4,114 cases in 2022, accounting for 18.8% of total registered cases across both sexes. 1

Northwest Syria is home to approximately 5.1 million people, more than two-thirds of whom are internally displaced, and where hostilities continue. The Syrian Ministry of Health withdrew from this region early in the conflict, 7 and health facilities are therefore supported by nongovernmental organizations (NGOs), which rely on funding either from private or from external donors. These donors primarily focus on covering the costs of preventative care (e.g., vaccination) to cover the greatest needs, which leaves cancer care as well as other high-cost conditions with insufficient funding and level of service. Few NGOs have the funds to meet cancer needs in this area, leaving cancer services substantially underfunded.

The provision of cancer care in the region is primarily managed by the Syrian American Medical Society (SAMS), a humanitarian aid organization providing health care to those affected by the Syrian conflict, which since 2020 has mostly been in northwest Syria where it contributes to around 15% of health services. SAMS offers a range of oncology services for the most prevalent types of cancer, such as breast, colorectal, leukemia, and lymphoma. In the cases where care is unavailable, patients must seek permission to access treatment in Turkey, bearing the cost and travel burdens associated with this option. 8 The earthquake that struck northwest Syria and southern Turkey on February 6, 2023, further complicated border crossings for referred oncology patients. A study in 2022 analyzed data of 1,654 pathology specimens recorded at Al-Rai Hospital pathology laboratory in Aleppo governorate 9 and found that breast cancer accounted for 20.2% of overall cancer cases in northwest Syria. This study highlighted the need for further investigations to assess the impact of conflict on cancer care.

Given these challenges, our study aims to explore the clinical characteristics, management, and outcomes of female patients diagnosed with breast cancer in northwest Syria.

## Methods

We conducted a retrospective descriptive analysis of a cohort of breast cancer cases by including all female breast cancer patients seen at the SAMS-supported Idlib Oncology Center between March 2017 and January 2022. This center is the only health facility in northwest Syria that provides a range of oncology services, including diagnosis, histopathology, surgical procedures, chemotherapy, and hormonal therapy.

### Study Setting

Northwest Syria, which includes parts of Idlib and Aleppo governorates. The population is estimated to be 5.1 million people, of whom two-thirds are internally displaced from other areas of Syria. Two million live in camps. 10 Access to health care is challenging due to insecurity, challenges around travel, associated costs of travel even where services are free, and overcrowded/underfunded facilities. More than 90% of people in this area live in poverty. 11

### Data Collection

Our dataset included routinely collected clinical data gathered from paper-based medical records and digitized with the assistance of medical students (A.H.), (A.A.), and (N.A.). The data included patient characteristics and medical history, encompassing preexisting conditions (e.g., diabetes and hypertension), as well as family medical background. Reproductive history included details about menopausal status, pregnancies, number of births, and breastfeeding. Clinical parameters included clinicopathological features such as cancer grade, stage, type, and metastasis. Hormone receptor statuses, including the TNBC (triple-negative breast cancer) test, were used, and histopathology was employed to categorize cancer molecular subtypes and histological classifications. Details on the received treatment were documented. The data covered information on surgical procedure details, endocrine therapy details (medication type, dates, ovarian suppression methods), chemotherapy (start dates, regimen used), and radiotherapy. As for treatment outcomes, instances of relapse were recorded along with the associated dates. conflict-related stressors included details about displacement status, exposure to chemical weapons, and the emotional strain from losing family members due to conflict.

### Descriptive Analysis

Data were extracted from case notes and entered into R statistics software ( https://www.R-project.org/ ) for analysis. Continuous data were summarized by means (standard deviations [SDs]) and medians (interquartile ranges [IQRs]), and categorical data were summarized using proportions (with percentages). When data were missing, the proportions were calculated using the number of participants with available data for each variable as the denominator.

### Statistical Analysis

We performed a least squares regression analysis to investigate the impact of conflict-related factors on survival outcomes. Three conflict-related variables were considered in this analysis: exposure to chemical weapons (whether individuals had been exposed), displacement status (comparing residents to internally displaced individuals), and the loss of a family member due to conflict.

### Ethical Considerations

The study was exempted from ethical approval by the ethics committee at Yale University (HIC# 2000031295). Participation in the study was voluntary. All patients were informed about the study purpose and provided their verbal consent to the data collection team. All data underwent anonymization before analysis, ensuring the removal of any identifiable information.

## Results

Data from 192 patients were included. Demographics, risk factors, diagnosis, and treatment details are outlined in Table 1 . The median age at diagnosis was 45.5 years (IQR: 40–56), with postmenopausal women accounting for 64 of 146 (43.8%) patients. Of 108 patients, 61 (56.5%) were internally displaced.

**Table 1:** Demographic characteristics

**Age, weight, and height (** ***N*** ** = 192)**	**Median**	**IQR**
Age at diagnosis (y)	45.5	40–56
Body mass index (BMI) at diagnosis	27.3	25.8–29
**Medical history (** ***N*** ** = 192)**	**No.**	**%**
Previous cancer	8	4.2
Family history of breast or ovarian cancer	14	7.3
Family history of other cancers	27	14.0
**Comorbidities (** ***N*** ** = 192)**	**No.**	**%**
Diabetes mellitus type II	19	9.8
Hypertension	26	13.5
Others	18	9.3
**Marital status at diagnosis (** ***N*** ** = 107)**	**No.**	**%**
Single	7	6.5
Married	77	72.0
Widowed	18	16.8
Divorced	5	4.7
**Menopausal status at diagnosis (** ***N*** ** = 146)**	**No.**	**%**
Premenopausal	82	56.2
Postmenopausal	64	43.8
Breastfeeding status ( *N* = 140)	68	48.6
**Reproductive history**	**Median**	**IQR**
No. of births ( *N* = 179)	5	3–7
No. of pregnancies ( *N* = 180)	6	4–9
**Conflict-related stressors**	**No.**	**%**
Displaced due to conflict ( *N* = 108)	61	56.5
Exposed to chemical weapons ( *N* = 107)	7	6.5
Lost a family member in conflict ( *N* = 132)	36	27.3

Abbreviations: IQR, interquartile range.

### Staging

Most patients, 156 (81.3%), were diagnosed with invasive ductal carcinoma (IDC), confirmed through the examination of biopsies at the pathology department attached to the center. Grade data for 147 patients revealed 66.0% as grade 2 and 31.3% as grade 3. The majority of patients presented at stages II and III (34.8 and 59.0%, respectively). Further details can be found in Table 2 .

**Table 2:** Clinical characteristics

**Receptors tests**	**No.**	**%**
Estrogen receptor positive ( *N* = 178)	132	74.2
Progesterone receptor positive ( *N* = 177)	113	63.8
TNBC ( *N* = 178)	21	11.8
**Clinical investigation**	**No.**	**%**
Palpable tumor ( *N* = 104)	94	90.4
Palpable lymph nodes ( *N* = 110)	80	72.7
**Histopathology (** ***N*** ** = 192)**	**No.**	**%**
Ductal carcinoma in situ	3	1.6
Invasive ductal carcinoma	156	81.3
Invasive lobular carcinoma	24	12.5
Other	9	4.7
**Grade (** ***N*** ** = 147)**	**No.**	**%**
1	4	2.7
2	97	66.0
3	46	31.3
**Stage (** ***N*** ** = 161)**	**No.**	**%**
I	4	2.4
II	56	34.8
III	95	59.0
IV	6	3.7

Abbreviation: TNBC, triple-negative breast cancer.

### Management

One hundred eighty-five out of 192 patients (95.8%) underwent surgery: 96.6% had a mastectomy and 3.4% had a lumpectomy. One hundred seventy-five out of 192 (90.7%) patients received chemotherapy. Of 150 patients who received chemotherapy, 96 (64.0%) completed treatment, 21 (14.0%) had interruptions due to conflict, and 33 (22.0%) were still on treatment at the time of data collection. Of 152 patients, 103 (53.4%) required radiotherapy, but only 50 (48.5%) received it; others did not for reasons noted in Table 3 .

**Table 3:** Treatment characteristics

**Treatment procedure**	**No.**	**(%)**
Surgery ( *N* = 185)	185.0	100.0
Chemotherapy ( *N* = 192)	175.0	90.7
Ovarian suppression ( *N* = 187)	20.0	10.7
Endocrine therapy ( *N* = 127)	105.0	54.6
Radiotherapy ( *N* = 148)	50.0	33.8
**Treatment technique and medication**	**No.**	**%**
**Surgery type (** ***N*** ** = 178)**		
Mastectomy	172.0	96.6
Lumpectomy	6.0	3.4
**Chemotherapy drug (** ***N*** ** = 173)**		
AC-T	138.0	78.0
TC	35.0	20.2
TCHP	2.0	1.2
**Endocrine medication (** ***N*** ** = 127)**		
Aromatase inhibitor	34.0	26.8
Tamoxifen	71.0	55.9
Did not need endocrine therapy	22.0	17.3
**Completion of chemotherapy (** ***N*** ** = 150)**		
Yes	96.0	64.0
In therapy	33.0	22.0
Interrupted	21.0	14.0
**Treatment intervals (d)**	**Median**	**IQR**
Between the appearance of syndromes and diagnosis ( *N* = 185)	100.5	25.7–309.5
Between the diagnosis and surgery ( *N* = 174)	14.5	7–20.2
Between the surgery and chemotherapy ( *N* = 175)	35.5	18.7–58.7
Between the surgery and radiotherapy ( *N* = 45)	230	187.5–280.7
**Post-op findings**	**Median**	**IQR**
Tumor size (cm), *N* = 106	4.0	3–6
No. of removed lymph nodes ( *N* = 165)	15.0	10–19
No. of positive lymph nodes removed ( *N* = 168)	4.0	1–8
**Access to radiotherapy**	**No.**	**%**
Patients who needed radiotherapy ( *N* = 152)	103.0	53.4
Reason for not getting the radiotherapy ( *N* = 32)		
Still in chemotherapy	13.0	40.6
Waiting access authorization	7.0	21.9
Refused to receive the treatment	4.0	12.5
Financial burden	3.0	9.4
For not allowing companion crossing the border	1.0	9.1
Dropped from treatment	2.0	6.3
Pregnancy	1.0	3.1
Death unrelated to cancer	1.0	3.1
**Completion of chemotherapy (** ***N*** ** = 150)**		
Yes	96.0	64.0
Still in therapy	33.0	22.0
Interrupted	21.0	14.0
**Treatment outcome**	**No.**	**%**
Relapsed ( *N* = 189)	9.0	4.8
Confirmed mortality ( *N* = 120)	13.0	10.8

Abbreviations: AC-T, doxorubicin and cyclophosphamide followed by taxane; IQR, interquartile range; TC, docetaxel and cyclophosphamide; TCHP, docetaxel, carboplatin, trastuzumab, and pertuzumab.

Of 185 patients who underwent surgery, the median tumor size was 4.0 cm (IQR: 3–6). Data on positive lymph nodes for 168 patients showed a median of 4 (IQR: 1–8). Distant metastases were identified in 30 (16.2%) of 185 patients.

### Timings of Treatment

The median interval between symptom onset and diagnosis was 100.5 days (IQR: 25.7–309.5); from diagnosis to surgery, it was 14.5 days (IQR: 7–20.2); and from surgery to chemotherapy, it was 35.5 days (IQR: 18.7–58.7). On the other hand, the median from surgery to radiotherapy was 229 days (IQR: 181.0–279.5).

### Statistical Analysis

Exposure to chemical weapons did not show a statistically significant effect on survival ( *p* = 0.4092; 95% CI: −0.1266 to 0.3084). Among the 107 individuals surveyed, 7 (6.6%) reported exposure to chemical weapons. Similarly, losing a family member during the conflict, reported by 36 out of 132 individuals (27.3%), did not significantly influence survival ( *p* = 0.1618; 95% CI: −0.02888 to 0.1706).

In contrast, displacement status was significantly associated with survival outcomes. Among 108 individuals, 61 (56.5%) reported being displaced due to conflict. The regression analysis demonstrated a statistically significant relationship ( *p* = 0.0038; 95% CI: 0.05064–0.2570).

## Discussion

This study is the first to provide insights into the characteristics and outcomes of breast cancer in conflict-affected northwest Syria, where the health care system has been severely compromised by ongoing and escalating armed conflict. Cancer is the second leading cause of mortality (10%) in Syria among noncommunicable diseases, right after cardiovascular diseases (25%). 12 There is a notable lack of studies on cancer within the country, particularly in conflict-affected areas such as northwest Syria.

Early-stage diagnosis of breast cancer significantly increases the 5-year survival rate; however, conflict and displacement appear to hinder early detection in several ways. These barriers include limited availability of health education and awareness among the population, 13 continued attacks on health care facilities, unstable living conditions, cultural stigma, and the scarce availability of diagnostic modalities. 14 These factors have exacerbated the situation, likely contributing to the high proportion of late-stage disease diagnoses and the correspondingly high rate of mastectomies observed in our study. In a review of breast cancer patients at Al-Bairouni Hospital in Damascus, it was found that less than 5% of cases were diagnosed at screening, which suggests inadequate coverage or access. 15 They found that 31% were stage II at presentation and 42% were stage III, whereas in our study, 34.8% were stage II and 59% were stage III, indicating an even worse cancer care reality in northwest Syria. Notably, 19% in their study were stage IV compared to 3.7% in our study. Studies conducted among Syrian refugees in neighboring countries have identified similarly late-stage presentations, 16 citing the silent nature of breast cancer in its early stages and the challenge of accessing comprehensive medical care in humanitarian settings. 17

Internally displaced individuals due to conflict encounter additional vulnerabilities compared to those experienced by other vulnerable populations in Syria to accessing timely cancer care, such as interrupted treatment, lack of continuity in care, and more difficulty in access to medical services. These issues are sometimes exacerbated by differences in social norms and traditions, particularly for women, due to displacement from different provinces or urban–rural contrasts. 18 These factors contribute to worsened survival outcomes compared to nondisplaced residents. Our study found a statistically significant association between displacement status and survival outcomes ( *p* = 0.0038), supporting the broader literature's conclusion that internal displacement negatively impacts cancer prognosis. 19 20

Our findings indicate that once a diagnosis is established, access to relatively inexpensive oncology treatments, such as surgery, is fairly rapid; the median time from diagnosis to surgery was 14.5 days (IQR: 7–20.2). The interval from surgery to systemic therapy/chemotherapy in our study was also relatively short at 35.5 days (IQR: 18.7–58.7). The chemotherapy regimens included AC-T (doxorubicin and cyclophosphamide followed by taxane), TC (docetaxel and cyclophosphamide), and TCHP (docetaxel, carboplatin, trastuzumab, and pertuzumab). Additionally, hormonal therapies such as aromatase inhibitors and tamoxifen were administered. These medications, most of which are listed on the WHO Model List of Essential Medicines (EML), 20 were procured by SAMS or provided through private donors rather than being supplied by United Nations (UN) agencies or institutional donors.

The expensive costs of cancer treatments tend to influence donor priorities, potentially leading to underfunding of essential cancer medications in conflict zones. 13 Implementing resource-stratified guidelines by international agencies was recommended to address these challenges. 13 In this approach, breast cancer patients can be managed effectively in resource-limited settings, 21 including basic surgery, affordable chemotherapy, and hormonal therapy. Such an option is far better than leaving the disease untreated. 13 Although these treatments are relatively affordable, many patients in northwest Syria face significant financial barriers without external support, which limits their access to necessary care. 22

We observed extended durations to access radiotherapy treatment, with a median delay of 229 days (IQR: 181.0–279.5). Notably, radiotherapy is not available in northwest Syria. Cancer patients in the region are referred to Turkey when such a treatment is indicated. The referral process involves submitting a detailed medical report where a panel of experts evaluates the case. Nonurgent cases, including many breast cancer patients, can face prolonged waiting times for a decision. In our study, 7 out of 32 patients were still waiting for access authorization at the time of data collection. In 2021, 1,700 cancer patients were referred to Turkey, a 26% decline from 2019, exacerbated by COVID-19 restrictions and economic pressures on Turkey's health care system. 8 Following the February 2023 earthquake, cross-border referral services were further disrupted, as oncology centers in southern Turkey suffered some earthquake damage, which critically impeded cancer patients' access to oncology services available in Turkey. 23 Even when referrals were possible, 3 out of 32 patients were unable to pursue treatment due to additional financial burdens. Even when therapy is free, the availability of free lodging and accommodations in Turkey is limited and cannot cover all patients, leaving many to bear these costs themselves. One patient refused to travel without a family member's support, which was not allowed by border crossing. These challenges add significant psychological strain on patients.

Given these barriers to accessing advanced therapy abroad, early recovery efforts should focus on strengthening and localizing oncology health care services within northwest Syria. 24 This requires investments in infrastructure, including appropriate equipment for diagnosis and radiotherapy. Additionally, building capacity through specialized oncology medical education and accreditation is essential. Addressing these needs will make cancer treatment more accessible locally, gradually reducing reliance on cross-border referrals.

Our study did not find a statistically significant association between the loss of a family member due to conflict and treatment outcomes in breast cancer patients. However, existing literature highlights the crucial role of social support, particularly from family and close friends, in enhancing the quality of life and treatment outcomes for cancer patients. Research shows that social support can mitigate feelings of distress and isolation, contributing significantly to the emotional and mental well-being of cancer patients throughout the treatment process. 25 For individuals undergoing cancer treatment, high levels of optimism and social support are linked to better mental health outcomes, including reduced anxiety and depression, as these elements help patients manage the stress associated with advanced cancer. 26 Similarly, in the context of cancer treatment, social support has been shown to enhance resilience and improve patients' capacity to cope with treatment demands, ultimately improving both immediate and long-term quality of life. 26

To explore this further, we conducted a separate qualitative study on patients from the same sample group in northwest Syria. Findings from this study align with existing literature, demonstrating that peer support, family encouragement, and faith play vital roles in helping breast cancer patients cope with their diagnosis amid conflict, emphasizing the importance of accessible, localized support networks. 27

We also did not find a significant association between exposure to chemical weapons and treatment outcomes. Notably, our sample included only a small number of patients ( *N* = 7) who were exposed to chemical weapons, potentially limiting the ability to yield a meaningful statistical finding. Interestingly, three of these patients were subjected to the same chemical attack that targeted the city of Khan Shaykhoun on April 4, 2017, where sarin (GB, O-isopropyl methylphosphonofluoridate) or a sarin-like substance was utilized by the Syrian government, as confirmed by the Organisation for the Prohibition of Chemical Weapons (OPCW). 28

The association between chemical weapons and cancer development remains poorly understood, as conducting such studies is particularly challenging. To date, no study has confirmed the carcinogenicity of sarin. A rare study involving human volunteers was conducted by the U.S. Army Chemical Corps to evaluate the impact of low-dose chemical warfare agents, including sarin. 29 In this study, no consistent pattern of increased cancer risk associated with sarin was identified over 20 years. However, the study had relatively low statistical power and was only able to detect large differences. From the limited studies available, sarin exposure can alter gene expression. 30 Additionally, a rare hemoglobin variant known as Hb Iraq-Halabja 31 was first reported in a patient from Halabja, a Kurdish city in northern Iraq, which was subjected to a sarin-included chemical weapon attack in 1988 by the Iraqi government. However, these mutations have not been firmly linked to carcinogenesis. 32 Finally, there is sufficient evidence to suggest that neurotoxic effects due to sarin exposure may be exacerbated by various factors, including heat and stress. 33 Such conditions were also faced by those affected by sarin in our study, who suffered from displacement and were living in camps.

## Strengths and Limitations

A key strength of this study is the comprehensive data collection from the Idlib Oncology Center, the primary oncology facility in northwest Syria, allowing detailed capture of patient demographics, clinical characteristics, and treatment pathways. Reliance on histopathological evaluations for diagnosis is another strength. However, as a retrospective study, it relies on existing medical records, which may have inconsistencies or incomplete data and lack long-term follow-up of patient outcomes. Variability in laboratory quality control may also impact the accuracy of receptor testing. Additionally, the study's focus on a single center and the absence of a cancer registry or surveillance system in Syria limit the generalizability of the findings to other regions or similar conflict zones.

## Conclusion

This study highlights numerous barriers to accessing breast cancer care in northwest Syria, driven by ongoing conflict, repeated displacement, and limited health care resources, further impacted by reduced cross-border referrals for advanced treatments. Addressing these challenges requires a comprehensive approach, including early screening, awareness programs, and the localization of oncology services with investment in radiotherapy infrastructure in northwest Syria.

NGOs, such as SAMS, have been pivotal in filling health care gaps left by the disrupted health system. Their contributions include establishing oncology centers and providing essential cancer treatments. Beyond these efforts, their role extends to advocacy for policy changes, securing international support, and developing low-cost treatment models tailored to conflict-affected, resource-limited environments.

Our study recommends that donors should increase their funding for secondary and tertiary health care, ensuring a steady supply of essential oncology medications from the WHO EML. This support should also extend to medical education initiatives, focusing on training health care professionals in specialized oncology fields. To strengthen data quality and guide future interventions, health clusters operating in conflict zones should prioritize establishing cancer registries and surveillance systems.

Further research is needed to establish effective, accessible treatment protocols suited for populations living in conflict. It is also essential to understand the broader cancer burden in Syria, focusing on high-mortality cancers like cervical and ovarian cancer, which often have poor outcomes when diagnosed late and lack routine screening in conflict-affected areas. Additionally, studies should examine the impact of ongoing conflict on continuity of care and treatment accessibility in resource-limited settings. Such research will aid in developing sustainable, context-specific cancer care models and alternative approaches suited to low-resource environments like northwest Syria, ultimately improving health outcomes for affected populations.
